# Evaluating the Reproducibility of Single-Cell Gene Regulatory Network Inference Algorithms

**DOI:** 10.3389/fgene.2021.617282

**Published:** 2021-03-22

**Authors:** Yoonjee Kang, Denis Thieffry, Laura Cantini

**Affiliations:** Computational Systems Biology Team, Institut de Biologie de l’Ecole Normale Supérieure, CNRS UMR 8197, INSERM U1024, Ecole Normale Supérieure, Paris Sciences et Lettres Research University, Paris, France

**Keywords:** biological networks, scRNA-seq, single-cell, transcriptome, network inference, network theory, reproducibility

## Abstract

Networks are powerful tools to represent and investigate biological systems. The development of algorithms inferring regulatory interactions from functional genomics data has been an active area of research. With the advent of single-cell RNA-seq data (scRNA-seq), numerous methods specifically designed to take advantage of single-cell datasets have been proposed. However, published benchmarks on single-cell network inference are mostly based on simulated data. Once applied to real data, these benchmarks take into account only a small set of genes and only compare the inferred networks with an imposed ground-truth. Here, we benchmark six single-cell network inference methods based on their reproducibility, i.e., their ability to infer similar networks when applied to two independent datasets for the same biological condition. We tested each of these methods on real data from three biological conditions: human retina, T-cells in colorectal cancer, and human hematopoiesis. Once taking into account networks with up to 100,000 links, GENIE3 results to be the most reproducible algorithm and, together with GRNBoost2, show higher intersection with ground-truth biological interactions. These results are independent from the single-cell sequencing platform, the cell type annotation system and the number of cells constituting the dataset. Finally, GRNBoost2 and CLR show more reproducible performance once a more stringent thresholding is applied to the networks (1,000–100 links). In order to ensure the reproducibility and ease extensions of this benchmark study, we implemented all the analyses in scNET, a Jupyter notebook available at https://github.com/ComputationalSystemsBiology/scNET.

## Introduction

Biological systems are inherently complex, in particular because of the emergent phenotypic properties arising from the interaction of their numerous molecular components. Characterizing genotype to phenotype connections and pathological deregulations thus requires to identify the biological macromolecules involved (e.g., genes, mRNAs, proteins), but also how these interact in a huge diversity of cellular pathways and networks (Barabási and Oltvai, 2004).

In the post-genomic era, biological networks have been extensively exploited to investigate such complex interactions among biological macromolecules (Barabási et al., 2011; Sonawane et al., 2019; Silverman et al., 2020). Network-based studies brought crucial insights into cell functioning and diseases (Basso et al., 2005; Margolin et al., 2006; Ideker and Sharan, 2008). A network is a graph-based representation of a biological system, where the nodes represent objects of interest (e.g., genes, mRNAs, proteins), while the edges represent relations between these objects (e.g., gene co-expression, or binding between two proteins). Different approaches can be used to reconstruct biological networks. Here, we focus on data-driven methods, which infer networks from gene expression data with the help of reverse engineering techniques (Sonawane et al., 2019).

Network inference algorithms were first proposed to extract information from bulk gene expression data, and their development has been an active area of research for more than 20 years (Barabási et al., 2011; The DREAM5 Consortium et al., 2012; Verny et al., 2017; Sonawane et al., 2019; Silverman et al., 2020). With the advent of single-cell RNA sequencing (scRNA-seq), we started to gather transcriptomic data from individual cells, enabling proper studies of their heterogeneity. However, the analysis of scRNA-seq data comes with a variety of computational challenges (e.g., small number of sequencing reads, systematic noise due to the stochasticity of gene expression at single-cell level, dropouts) that distinguish this data type from its bulk counterpart. For this reason, network inference methods originally developed for bulk gene expression data may not be suitable for data generated from single cells. The development of network inference algorithms has thus recently undergone a strong shift towards the design of methods targeting single-cell data (Fiers et al., 2018).

Two benchmarks of single-cell network inference methods have been published (Chen and Mar, 2018; Pratapa et al., 2020). Both works evaluate network inference algorithms by comparing the inferred network with a ground-truth. These works are also mostly focused on simulated data and they apply a strong filtering on genes (leaving only 100–1,000 genes for network inference). Chen and Mar (2018) considered five methods targeting bulk data and three methods specifically designed for single-cell data. More recently, Pratapa et al. (2020) focused on 12 methods designed for single-cell data. Both benchmarks concluded that the overall performances of all methods were quite disappointing, and that network inference remains a challenging problem.

Here, we evaluate network inference algorithms based on their reproducibility, i.e., their ability to infer similar networks once applied to two independent datasets for the same biological condition (e.g., two independent scRNA-seq datasets obtained from colorectal tumors). The rationale behind this comparison is that, if the two independent datasets are profiled from the same biological condition (e.g., colorectal cancer, CRC) involving the same cell types, we can expect that the regulatory programs underlying them should strongly overlap. As a consequence, a good network inference algorithm should infer highly overlapping networks when applied to single-cell datasets profiled from the same biological condition. We selected six algorithms spanning the main network inference formulations that do not require an ordering of the cells according to pseudo-time, and we tested the reproducibility of the inferred networks in three biological systems: human retina, T-cells in CRC and human hematopoiesis. Differently from previous benchmarks, we only applied a soft filtering on genes, thus testing the algorithms based on their performances to infer networks involving from 6,000 to 12,000 nodes/genes.

From our benchmark, once an high number of links is taken into account (100,000), GEne Network Inference with Ensemble of Trees (GENIE3) results to be the most reproducible algorithm and, together with GRNBoost2, show the highest intersection with ground-truth biological interactions. GRNBoost2 and Context Likelihood of Relatedness (CLR) have instead better performances for low link numbers (1,000–100). In order to ensure the reproducibility and ease extensions of this benchmark study, we implemented all the analyses in a Jupyter notebook, called scNET and available at https://github.com/ComputationalSystemsBiology/scNET.

## Materials and Methods

### Benchmarked Single-Cell Network Inference Algorithms

Starting from the exhaustive collection of single-cell network inference algorithms presented in Chen and Mar (2018) and Pratapa et al. (2020), two main categories of methods can be distinguished. Some methods interpret scRNA-seq as time-course expression data, where the pseudo-time corresponds to the time information. These methods are frequently based on Ordinary Differential Equations (ODEs) and are relevant for biological systems undergoing dynamic transcriptional changes (e.g., scRNA-Seq performed on differentiating cells) (Matsumoto et al., 2017). In contrast, other methods do not use pseudo-time information to infer networks. These methods generally use statistical measures (e.g., correlation, mutual information) to infer regulatory connections and are thus better suited for transcriptomic data not affected by strong dynamical processes (e.g., retina cells in normal state).

Testing reproducibility strictly requires the availability of two independent scRNA-seq datasets reflecting the same biological condition and presenting as few as possible technical variations. Indeed, the presence of technical variations due to the sequencing or experimental procedures could drastically impact the outcome of our comparison. In this respect, finding independent scRNA-seq datasets reflecting dynamic transcriptional changes, generated with the same experimental procedure, is really challenging. We thus decided to focus our benchmark study on network inference methods that do not use the pseudo-time information. In addition, only algorithms provided in R or Python code are here taken into account. Six single-cell network inference methods are thus considered in this evaluation: GENIE3 (Huynh-Thu et al., 2010), GRNBoost2 (Moerman et al., 2019), PPCOR (Kim, 2015), Partial Information Decomposition and Context (PIDC; Chan et al., 2017), CLR (Faith et al., 2007), and GeneNet (Opgen-Rhein and Strimmer, 2007). All the methods selected for this benchmark were originally designed for bulk data and they span the main mathematical formulations of network inference, as described in The DREAM5 Consortium et al. (2012). Of note, GENIE3, GRNBoost2 and PIDC are also the best performing in the single-cell benchmark of Pratapa et al. (2020).

GEne Network Inference with Ensemble of Trees (Huynh-Thu et al., 2010) is a tree-based network inference method. For each gene g_i_ in the expression dataset, GENIE3 solves a regression problem, determining the subset of genes whose expression is the most predictive of the expression of g_i_. This method was the best performing algorithm in the DREAM4 In Silico Multifactorial challenge (Greenfield et al., 2010). GENIE3 requires in input the scRNA-seq expression matrix and a list of Transcription Factors (TFs). In our tests the list of human TFs provided in input corresponds to the intersection between the expressed genes and those annotated as encoding TFs by Chawla et al. (2013). The output of GENIE3 is a weighted network linking TFs with predicted target genes. The weight associated with each link corresponds to its Importance Measure (IM), which represents the weight that the TF has in the prediction of the level of expression of the target gene. We run GENIE3 from the Arboreto library (Moerman et al., 2019) using default parameters.

GRNBoost2 (Moerman et al., 2019) has been developed as a faster alternative to GENIE3. It is thus based on a regression model, using a stochastic gradient boosting machine regression. The inputs and outputs of GRNBoost2 have the same structure of those of GENIE3. Both GRNBoost2 and GENIE3 are part of the SCENIC workflow (Aibar et al., 2017). We run GRNBoost2 from the Arboreto library (Moerman et al., 2019) using default parameters.

PPCOR (Kim, 2015) infers the presence of a regulatory interaction between two genes by computing the correlation of their expression patterns. To control for possible indirect effects, partial correlation is used instead of a simple correlation, where partial correlation is a measure of the relationship between two variables while controlling for the effect of other variables. The only input of PPCOR is the expression matrix. The output of PPCOR is a weighted network, where all links are weighted based on the partial correlation between the expression values of the linked nodes/genes.

Partial Information Decomposition and Context (Chan et al., 2017) is based on concepts from information theory and uses partial information decomposition (PID) to identify potential regulatory relationships between genes. The only input of PIDC is the expression matrix and its output is a weighted gene-gene network.

Context Likelihood of Relatedness (Faith et al., 2007) is another commonly used approach based on concepts from information theory. The measure used by CLR to infer links in between genes is Mutual Information (MI). In contrast with other algorithms also based on MI, such as ARACNE (Margolin et al., 2006), CLR adjusts the link weights for the background distribution of the MI values to control for false positives interactions.

GeneNet (Opgen-Rhein and Strimmer, 2007) is a method for statistical learning of a high-dimensional causal network. The method first converts a correlation network into a partial correlation graph. Subsequently, a partial ordering of the nodes is established by multiple testing of the log-ratio of standardized partial variances.

To make the different network inference algorithms comparable, we applied the same thresholding to all of them, by keeping only the top K links (K = 100,000). For GeneNet, inferring less than 100,000 links, no filtering has been applied.

### Data Acquisition and Preprocessing

Fourteen public scRNA-seq datasets have been used for this benchmark (Table 1): Lukowski et al. (2019) and Menon et al. (2019) obtained by profiling human retina cells; Li et al. (2017) and Zhang et al. (2019) profiling T-cells in CRC; Hay et al. (2018) and Setty et al. (2019) profiling human hematopoiesis cells. See Table 1 for a complete description of these datasets. The hematopoiesis datasets were split according to their cell type of origin. Only those cell types reported in both studies by Hay et al. (2018) and Setty et al. (2019) were considered. We thus obtained a total of 10 scRNA-seq datasets in hematopoiesis spanning five cell types: HSC, CLP, Monocyte, Erythroblast, and Dendritic Cell.

**TABLE 1:** Datasets employed in this benchmark.

Data	Biological	Sequencing	Number of	Cell type	Associated	Number of genes
Name	context	technology	cells	annotation strategy	publication	after preprocessing
Menon	Human retina	10X Genomics	20,091	Manually curated marker genes	Menon et al., 2019	6,212
Lukowski	Human retina	10X Genomics	20,009	No annotation	Lukowski et al., 2019	6,212
Zhang	CRC T-cells	Smart-Seq2,	10,805	FACS sorted	Zhang et al., 2019	11,242
Li	CRC T-cells	HiSeq 2000 Illumina	375 cells (of which 35 T-cells)	Manually curated marker genes	Li et al., 2017	11,242
Hay	human hematopoiesis	10X Genomics	101,935	MarkerFinder ICGS	Hay et al., 2018	7,038
Setty	human hematopoiesis	10X Genomics	12,046	Sorted bulk hematopoietic populations	Setty et al., 2019	7,038

After downloading the data, we filtered the genes based on their total count number (<3 × 0.01 × number of cells), as well as on the number of cells in which they are detected (>0.01 × number of cells), as described in Aibar et al. (2017). The gene filtering is performed on each dataset independently. Then, for each biological condition (CRC T-cells, retina, and hematopoiesis), only the genes retained for both datasets were selected for network inference. The number of genes retained after filtering are reported in the last column of Table 1. Finally, the data were log2-normalized before applying the different network inference algorithms.

### Indexes Employed to Measure the Reproducibility of the Network Inference Algorithms

Percentage of intersection (perINT) and Weighted Jaccard Similarity (WJS) have been employed here to assess the reproducibility of the network inference algorithms. The percentage of intersection is used to detect the presence of links shared between two compared networks, while WJS takes into account the similarity of the weights associated with the links shared between the compared networks.

Given two networks N_1_ and N_2_ inferred respectively from scRNAseq datasets D_1_ and D_2_, and indicating as |*N*| the number of links in the network N, the perINT is computed as:

$$perINT\left(N_{1},N_{2}\right)=\frac{\left|N_{1}\cap N_{2}\right|}{min\left(\left|N_{1}\right|,\left|N_{2}\right|\right)},$$

while the WJS (Tantardini et al., 2019), is defined as

$$WJS\left(N_{1},N_{2}\right)=\frac{\sum_{i=1}^{\left|N\right|}min\left(w_{i}^{1},w_{j}^{2}\right)}{\sum_{i=1}^{\left|N\right|}max\left(w_{i}^{1},w_{j}^{2}\right)},$$

where *w*^1^,*w*^2^ are the vectors of weights associated with the links in common between N_1_ and N_2_.

In addition, to compare the inferred links to a ground-truth, we considered two additional scores: RcisTarget and Regulatory Circuit scores. We derived the RcisTarget score from the application of the RcisTarget tool (Aerts et al., 2010; Aibar et al., 2017). Given a network of TF-gene interaction, RcisTarget predicts candidate target genes of a TF by looking at the DNA motifs that are significantly over-represented in the surroundings of the Transcription Start Site (TSS) of all the genes that are linked to the TF. We here consider the links validated by RcisTarget as ground-truth and we compare them with the inferred networks, by computing:

$$RcisTargetScore\left(N_{1}\right)=\frac{NumberLinks\in N_{1}\cap ValidatedByRcisTarget}{\left|N_{1}\right|}$$

In the case of the methods inferring links between all genes, a selection of links connecting TFs with possible target genes is performed before computing the RcisTaget score.

The Regulatory Circuits score instead is obtained by computing the intersection between an inferred network and tissue-specific regulatory circuits from http://www.regulatorycircuits.org (Marbach et al., 2016). The regulatory circuits considered are the following: adult retina for retina, lymphocytes for CRC T-cells and CD34 stem cell derived for hematopoiesis. We here computed the Regulatory Circuits score for a network N_1_ as:

$$RegulatoryCircuitScore\left(N_{1}\right)=\frac{\left|N_{1}\cap AssociatedRegulatoryCircuits\right|}{\left|N_{1}\right|}$$

## Results

**FIGURE 1:**
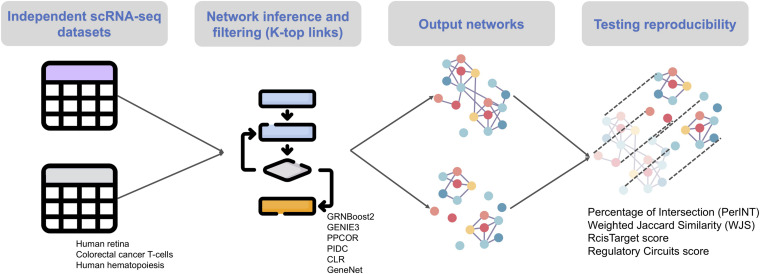
Summary of the workflow followed in this benchmark.

Based on previous works (Chen and Mar, 2018; Pratapa et al., 2020), we selected the six single-cell network inference algorithms that do not require an ordering of the cells according to pseudo-time (GENIE3, GRNBoost2, PPCOR, PIDC, CLR and GeneNet see section “Materials and Methods”) and we evaluated them based on their reproducibility, i.e., their ability to infer similar networks once applied to two independent datasets from the same biological condition (e.g., two independent scRNA-seq datasets of CRC). The reproducibility is measured based on the perINT and WJS indexes (see section “Materials and Methods”). In addition, we computed the intersection with two instances of ground-truth, based on the RcisTarget and on Regulatory Circuits scores (see section “Materials and Methods”). The evaluation is repeated across three biological conditions: human retina, T-cells in CRC and human hematopoiesis, for a total of 14 independent scRNAseq datasets. See Figure 1 for an overview of the benchmark workflow.

While in previous benchmarks (Chen and Mar, 2018; Pratapa et al., 2020), a low number of highly variable genes had been taken into account (100–1,000 genes), we here tested the ability of the algorithms to infer networks involving all expressed genes (see section “Materials and Methods” for details on the procedure used to filter genes). Indeed, filtering only the top 100–1,000 varying genes is a strong limitation. Restricting the nodes of the inferred network to a low number of genes is reasonable when a manually curated list of relevant genes is available (for example marker genes identified by wet-lab experiments). However, when such a list is not available, working only with the top 100–1,000 varying genes may overlook genes and interactions playing a key role in the regulatory programs of the biological system. We thus tested the various network inference algorithms once applied to scRNAseq datasets containing 6,000–11,000 genes.

In our test cases, PIDC failed to reconstruct networks for two main reasons: (i) the algorithms was slow, especially in the discretization step required to infer a network and (ii) the use of multivariate information measures impose to have a number of genes much lower than the number of cells, thus requiring to drastically filter out the starting set of genes. Overall, PIDC thus resulted to be more adequate to infer small networks (100–1,000 nodes/genes), which are not the focus of this work.

### Reproducibility in Human Retina

We applied GENIE3, GRNBoost2, PPCOR, CLR, and GeneNet to two independent scRNA-seq datasets of human retina, reported in Menon et al. (2019) and Lukowski et al. (2019) (see section “Materials and Methods”). After filtering, the two datasets span 6,212 common genes across a comparable number of cells: 20,091 in Menon versus 20,009 in Lukowski.

We thus inferred a total of ten networks. Details on the number of links before and after thresholding are provided in the Supplementary Table 1. We then evaluated the reproducibility of each algorithm by computing the perINT and the WJS between the networks inferred independently from the two datasets. While perINT is intended to test the amount of common links between the two networks, the WJS takes also into account the similarity of the weights associated with the common links.

**FIGURE 2:**
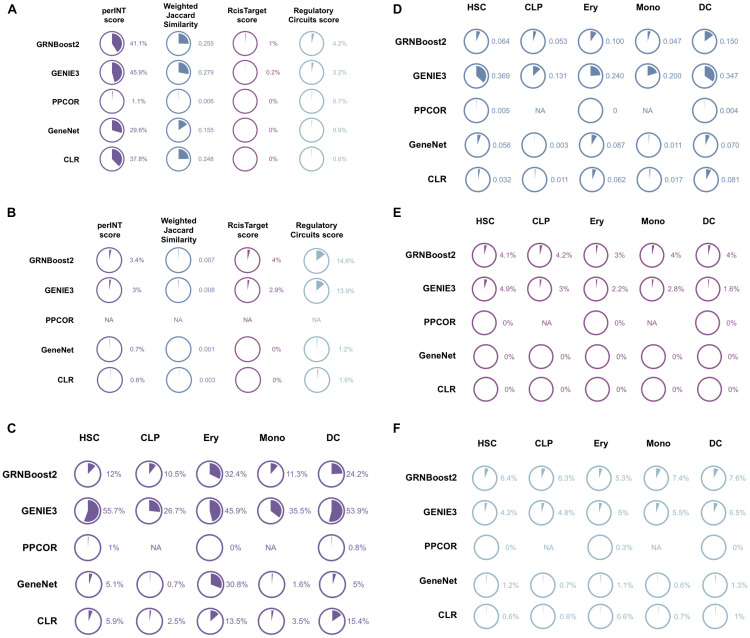
Reproducibility performances of the various network inference algorithms across the three biological contexts: human retina, colorectal cancer T-cells and human hematopoiesis. Panels **(A,B)** report the Percentage of intersection (perINT), Weighted Jaccard Similarity (WJS), RcisTarget score and Regulatory Circuits score obtained by each the benchmarked algorithm (GRNBoost2, GENIE3, PPCOR, CLR, and GeneNet) in human retina and colorectal cancer T-cells, respectively. Panels **(C–F)** summarize the performances of the same algorithms on the hematopoiesis datasets, with perINT **(C)**, WJS **(D)**, RcisTarget score **(E)**, and Regulatory Circuits score **(F)**.

As shown in Figure 2A, GENIE3 (45.9% perINT and 0.28 WJS) and GRNBoost2 (41.1% perINT and 0.25 WJS) are the algorithms showing the highest reproducibility, with GENIE3 performing slightly better. At the same time, in agreement with the results of the previous benchmarks, the intersection with the ground truth considered remains rather low, but higher for GRNBoost2 (1% RcisTarget score and 4.2% Regulatory Circuits score). Similar performances apply also for the other network inference methods.

### Reproducibility in Colorectal Cancer T-Cells

We further tested the performances of GENIE3, GRNBoost2, PPCOR, CLR, and GeneNet in CRC T-cells. The two datasets used in this case are taken from Zhang et al. (2019) and Li et al. (2017) (see section “Materials and Methods”), restricting the last dataset to only T-cells (see section “Materials and Methods”). After filtering, we obtained datasets composed of 11,242 common genes and a widely varying number of cells: 10,805 for Zhang, and 35 for Li.

We applied GENIE3, GRNBoost2, PPCOR, CLR and GeneNet independently to the two datasets (for details on the number of links before and after thresholding, refer to Supplementary Table 1). Of note, PPCOR has been excluded from this comparison, as it produced partial correlation values outside the range [-1;1] for the Li et al. dataset.

After computation of the perINT and WJS (Figure 2B), GENIE3 (3% perINT and 0.008 WJS) and GRNBoost2 (3.4% perINT and 0.007 WJS) emerged as the best performing methods. The reproducibility indexes are quite low in this test case, probably due to the low number of cells present in the Li dataset (35 cells). The RcisTarget and Regulatory Circuits scores reflecting the intersection with a ground-truth are also quite low for all algorithms, with GRNBoost2 showing better performances (4% RcisTarget score and 14.6% Regulatory Circuits score).

### Reproducibility in Human Hematopoiesis

Human hematopoiesis has been used as the third biological context for the comparison of GENIE3, GRNBoost2, PPCOR, CLR, and GeneNet. The hematopoiesis datasets were split according to the different cell types profiled: HSC, CLP, Monocyte, Erythroblast, and Dendritic Cell, obtaining a total of 10 scRNA-seq datasets. Networks were thus inferred on each cell type independently with GENIE3, GRNBoost2, PPCOR, CLR, and GeneNet, resulting in a total of 50 networks. Details on the number of links before and after thresholding are available in Supplementary Table 1. As for CRC T-cells, PPCOR produced networks composed of links with partial correlation higher than 1 and/or lower than -1 for some CLPs, and Monocytes. For this reason, we did not consider PPCOR in the reproducibility evaluation for these cell types.

The reproducibility was then tested for each cell type using the perINT and WJS indexes (Figures 2C,D). GENIE3 displayed the best performances with percentages of intersection of 26–56% and WJS at 0.13–0.37. Consistently with previous observations, the RcisTarget and Regulatory Circuits scores remain low for all cell types and all methods, with GRNBoost2 having slightly better performances than GENIE3 (approx. 2–4.2% and 4–7.6%, respectively) (Figures 2E,F).

### Stability With Respect to Link Thresholding in the Inferred Networks

In the previous experiments, the 100,000 top-ranked links have been taken into account for all methods, except GeneNet having less than 100,000 links (see section “Materials and Methods,” Supplementary Table 1). Here we test to which extent our conclusions, regarding the reproducibility of the benchmarked methods, are stable with respect to the number (K) of links retained in each network. We thus apply a more stringent filtering, considering an identical number (K) of top-ranked links of 10,000, 1,000, and 100 for all compared methods. GeneNet has been excluded from this analysis, as the number of its inferred links is lower than 1,000 in most of the cases. After thresholding, the intersection between the networks inferred from independent datasets from the same biological condition were evaluated, using the percINT and WJS as above.

**FIGURE 3:**
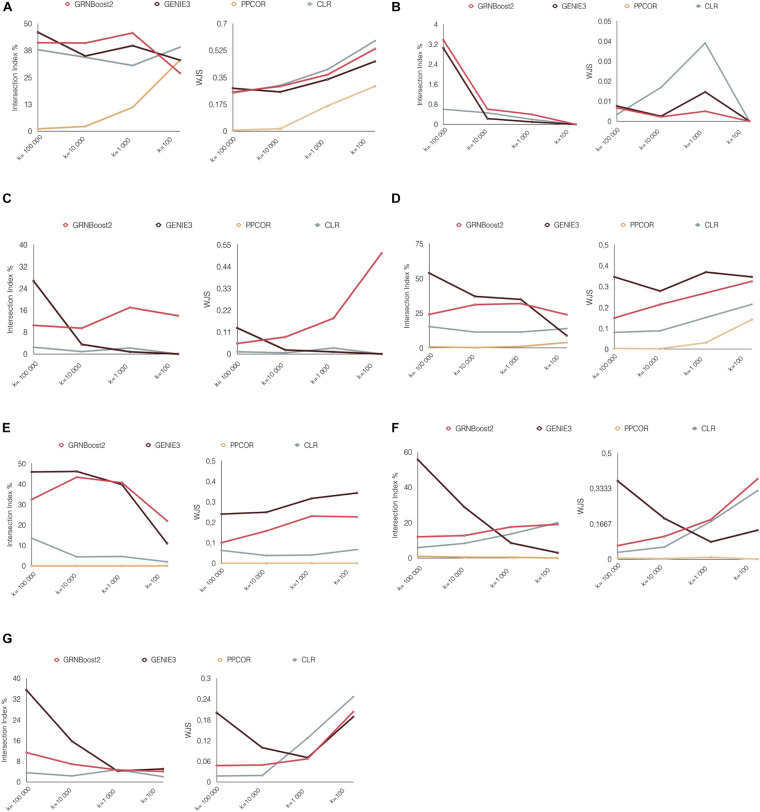
perINT and WJS according to different network thresholding. The perINT and WJS are reported for varying values of the threshold K on the network links: 100,000, 10,000, 1,000, and 100. The results are reported for all the tested datasets **(A)** retina, **(B)** CRC T-cells, **(C)** CLPs, **(D)** Dendritic cells, **(E)** Erythrocytes, **(F)** HSCs, and **(G)** Monocytes.

As shown in Figure 3, the performances of the various algorithms are quite heterogeneous once different thresholds (K) are considered. As observed in the previous sections, GENIE3 tends to have better performances for high K. However, for low numbers of links (K = 1,000 and 100), GRNBoost2 and CLR tend to predominate in most of the cases.

### Stability With Respect to Technical Variations in the Input Data: Number of Profiled Cells, Sequencing Platform, and Cell Type Annotation

In the experiments performed above, we tested the reproducibility of the network inference algorithms by using two independent datasets for each biological condition (e.g., human retina). A limitation of this approach comes from the technical differences between the protocols followed to generate these datasets: different sequencing platforms, different procedures used for the annotation of the cell types, and different number of cells. All these technical differences could impact our results.

To evaluate the stability of the results against technical variations, we used the largest dataset, from Menon et al. (2019), encompassing 20,091 cells. We splitted this dataset into two subsets, keeping the proportions of the various cell types constant. We then applied the five network inference algorithms independently to the two subsets, and we evaluated the reproducibility of the algorithms using perINT and WJS, as in the previous tests. To further assess the effect of the number of cells on network inference, we split the same scRNAseq dataset generated by Menon et al. (2019) three times to obtain couples of datasets encompassing decreasing numbers of cells: 10,000, 1,000, and 100. Note that for all these comparisons, the sequencing platform and/or the method/technique used to annotate the cells are identical for all subsets. PPCOR inferred networks for 10,000 and 1,000 cells, but failed at 100 cells (see Supplementary Table 2). Details on the number of links before and after thresholding (K = 100,000) are provided in the Supplementary Table 2.

**FIGURE 4:**
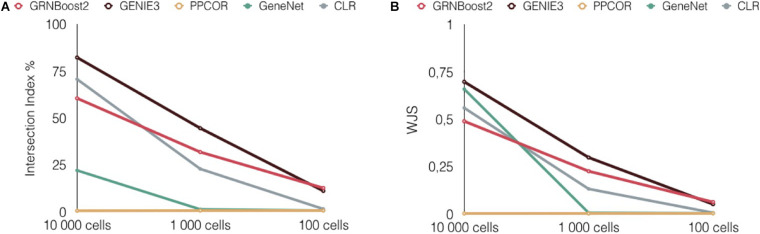
Stability of the network inference performances with respect to technical variations in the input data. Reproducibility scores of GRENBoost2 (red), GENIE3 (black) PPCOR (yellow), CLR (gray) and GeneNet (green) across different splittings of the Menon retina dataset. Panels **(A,B)** report the percentage of intersection (perINT) and Weighted Jaccard Similarity (WJS), respectively.

Overall, as shown in Figure 4, GENIE 3 emerged again as the best performing method in all cases. Of note, for low number of cells, a general decrease in reproducibility is observed for all network inference methods, which can be justified by a lower accuracy in the link estimation due to the low number of observations (cells).

### The scNET Jupyter Notebook

To foster the reproducibility of all the results and figures presented in this study, we implemented the corresponding code in a Jupyter notebook, available on GitHub, at the url https://github.com/ComputationalSystemsBiology/scNET, together with a Conda package containing all the required libraries. Importantly, scNET can be used to benchmark new network inference algorithms based on their reproducibility, or further test GENIE3, PPCOR, GRNBoost2, CLR, and GeneNet on user-provided datasets.

## Discussion

Starting from the benchmark of Pratapa et al. (2020), we evaluated the network inference algorithms from a complementary perspective by assessing their reproducibility. We were interested in assessing weither the algorithms would infer similar networks when applied to pairs of independent datasets from the same biological condition (e.g., T-cells in CRC). Our benchmark focused on real patient-derived data spanning three biological contexts: human retina, T-cells in CRC, and human hematopoiesis cells. We thus considered highly different biological contexts, going from cancer tissue, to isolated healthy immune cells, and to a mixture of normal retina cells combined in a single dataset. Importantly, we aimed at inferring networks involving a much higher number of genes compared to previous works.

In agreement with previous benchmarks, all network inference algorithms generated networks having low intersections with ground-truth. Of note, the ground-truth considered here, based on RcisTarget and regulatory circuits, is different and complementary to those used in previous benchmarks. This disappointing result might arise for different reasons, potentially adding up. Limitations can be present in the input data, as scRNAseq may not provide sufficient resolution for reliable network inference, and technical and experimental factors present in the input data might affect information content. Turning to the inference algorithm, limitations may arise from underlying statistical assumptions and the documented lack of uniqueness in the solution of the network inference problem. Finally, the ground-truth network considered here and in previous benchmarks may not be sufficiently comprehensive.

PPCOR provided weights outside the normal range of correlation values ([-1,1]) for datasets having less than 1,000 cells. Such inconsistencies are likely due to numerical problems arising when the input dataset encompasses many more genes than cells. PIDC was the algorithm that suffered the most when applied to high numbers of genes. Overall, for high link numbers (K = 100,000), GENIE3 consistently generated the most reproducible results across all the three biological contexts considered. Furthermore, its performances proved to be stable with respect to the single-cell sequencing platform, the cell type annotation system and the number of cells considered. Once a more stringent filtering is considered (K = 1,000 or 100), CLR and GRNBoost2 show better performances. However, even the best performing methods show reproducibility scores that are less than ideal (26–54% perINT and 0.1–0.3 WJS), indicating that further improvements are still needed in the design of network inference methods for scRNA-seq data.

We considered network inference methods that are highly heterogeneous. Some algorithms, as PPCOR and GeneNet, infer links between all possible couples of genes, while others, as GENIE3 and GRNBoost2, only infer links between TFs and possible target genes. We tried to make the inferred networks comparable by fixing the number of links in all networks to a certain value K, thus obtaining networks with the same density. However, in principle, methods inferring only TF-target links should have higher chances to be reproducible in our comparison. At the same time, once the links of PPCOR and GeneNet are restricted to only TF-target links, the dimension of the networks drastically decreases (sometimes empty networks are obtained).

The main limitation of this benchmark is the number of considered network inference algorithms. Future extensions of this study could include pseudotime-based network inference methods, once adequate datasets will become available. To date, available independent datasets relevant for pseudotime-based network inference algorithms (e.g., cells profiled during development stimulation) present too many experimental variations to be employed for a reliable evaluation of reproducibility. Of note, such extensions will be greatly facilitated by taking advantage of the Jupyter notebook (scNET) provided as Supplementary Material.

## Data Availability Statement

Publicly available datasets were analyzed in this study. The datasets for this study can be accessed from their associated publications (see Table 1). All the analyses are reproducible using the scNET Jupyter notebook available at https://github.com/ComputationalSystemsBiology/scNET.

## Author Contributions

LC designed the analysis. YK performed the analysis. LC and DT co-supervised the study. All authors contributed to the manuscript and approved the submitted version.

## Conflict of Interest

The authors declare that the research was conducted in the absence of any commercial or financial relationships that could be construed as a potential conflict of interest.
