# Supplementary material for: Evaluating the Reproducibility of Single-Cell Gene Regulatory Network Inference Algorithms
Source: Front Genet. 2021 Mar 22;12:617282. doi: 10.3389/fgene.2021.617282 (PMC8019823; doi:10.3389/fgene.2021.617282)
Supplement: Supplementary file 1 [file Data_Sheet_1.docx]

Supplementary Material

# Supplementary Tables

#
